# Investigation of left atrial mechanical function and left ventricular systolic and diastolic parameters in athletes performing resistance exercise and combined exercise

**DOI:** 10.1186/s12872-024-03908-w

**Published:** 2024-05-05

**Authors:** Ahmet Kurtoğlu, Alkame Akgümüş, Ahmet Balun, Engin Aydın, Ertuğrul Kurtoğlu, Bekir Çar, Nurettin Konar, Özgür Eken, Hadi Nobari

**Affiliations:** 1 Department of Coaching Education, Faculty of Sport Science, Bandirma Onyedi Eylul University, Band?rma/Bal?kesir, 10200 Türkiye; 2 Department of Cardiology, Medical Faculty, Bandirma Onyedi Eylul University, Bandırma, Balıkesir, 10200 Türkiye; 3 Department of Pediatrics, Zeynep Kamil Maternity and Children’s Disease Training and Research Hospital, University of Health Sciences, Istanbul, 34668 Türkiye; 4 Department of Cardiology, Medical Faculty, Malatya Turgut Ozal University, Battalgazi/Malatya, 44000 Türkiye; 5 Department of Physical Education and Sport Teaching, Faculty of Sport Sciences, Bandirma Onyedi Eylul University, Band?rma/Bal?kesir, 10200 Türkiye; 6 Department of Physical Education and Sport Teaching, Faculty of Sports Sciences, Inonu University, Malatya, 44000 Türkiye; 7 Faculty of Sport Sciences, University of Extremadura, Cáceres, 10003 Spain; 8 Department of Exercise Physiology, Faculty of Educational Sciences and Psychology, University of Mohaghegh Ardabili, Ardabil, 56199-11367 Iran

**Keywords:** Resistance exercise, Combined exercise, Echocardiography, Left atrial function, Systolic function, Diastolic function

## Abstract

Some individuals who go to fitness centers for various purposes perform resistance exercise (RE) alone, while others engage in combined exercise (CE) by including cardio exercises along with RE. Studying the effects of these two different training methods on left ventricular (LV) systolic and diastolic parameters and left atrial mechanical function is an important step toward understanding the effects of different types of exercise on cardiac function. This knowledge has significant implications for public health, as it can inform the development of targeted and effective exercise programs that prioritize cardiovascular health and reduce the risk of adverse outcomes. Therefore, the primary aim of this study is to comprehensively investigate the LV systolic and diastolic parameters of athletes who engage in RE and CE using ECHO, to contribute to the growing body of literature on the cardiovascular effects of different types of exercise. Forty-two amateur athletes aged between 17 and 52 were included in our study. The participants consisted of the RE (*n* = 26) group who did only resistance exercise during the weekly exercise period, and the CE group (*n* = 16) who also did cardio exercise with resistance exercises. After determining sports age (year), weekly exercise frequency (day), and training volume (min) in addition to demographic information of RE and CE groups, left ventricular systolic and diastolic parameters and left atrial functions were determined by ECHO. Findings from our study revealed that parameters including the left ventricular end-diastolic diameter (LVEDD) (*p* = .008), left ventricular end-diastolic volume (LVEDV) (*p* = .020), stroke volume index (SV-I) (*p* = .048), conduit volume (CV-I) (*p* = .001), and aortic strain (AS) (*p* = .017) were notably higher in the RE group compared to the CE group. Also left atrial active emptying volüme (LAAEV) of CE was higher than the RE group (*p* = .031). In conclusion, the cardiac parameters of the RE group showed more athlete’s heart characteristics than the CE group. These results may help to optimize the cardiovascular benefits of exercise routines while minimizing the potential risks associated with improper training.

**Supplementary Information:**

The online version contains supplementary material available at 10.1186/s12872-024-03908-w.

## Background

Cardiovascular diseases are the most common cause of mortality and morbidity today [1]. Sport is an important phenomenon in the rehabilitation and prevention of cardiovascular diseases. Chronic exercise causes some improvements and some morphological changes in the heart [2]. Athlete’s heart refers to electrophysiological, structural, and functional myocardial adaptations associated with continuous training stimuli without pathological significance [3]. Regular physical training leads to an increase in left ventricular (LV) cavity size and mass (LVM) due to increased wall thickness, with adaptations occurring in endurance athletes with the greatest volume and exercise intensity [4]. In general, in the athlete’s heart, an enlargement of the left and right ventricles by 10–15% and of the left ventricular wall thickness by 10–20% is observed [5]. Echocardiographic findings can help to identify adaptive and exercise-induced cardiac changes associated with underlying pathology. Researchers have been studying these changes in cardiac structure and function for many years, as they are important for understanding the effects of exercise on the heart [6].

The wide range of cardiovascular hemodynamic changes requires classification into two main categories: static and dynamic, with several differences depending on the sport. Resistance exercise is generally characterized by static, short, and strong muscle contractions. Static sports lead to an increase in systemic vascular resistance, systolic blood pressure (SBP), and heart rate (HR) to maintain cardiac output, which eventually manifests as concentric LV hypertrophy [7]. On the other hand, dynamic and endurance activities such as running are characterized by repeated contractions and relaxations of the large skeletal muscles. Dynamic exercise results in an increase in HR, an increase in stroke volume (SV), and an increase in SBP while decreasing systemic vascular resistance and diastolic blood pressure (DBP) to maintain adequate oxygen delivery [8].

Resistance exercise (RE), also known as weight or strength training, is a specialized form of conditioning to increase muscular strength, muscular endurance, and muscular power. In response to this type of training, both skeletal and cardiac muscles adapt to this condition, resulting in hypertrophy [9]. However, RE and endurance exercises lead to different changes in the heart. These changes in RE are characterized by an increase in LV wall thickness and no change in the diastole LV cavity [10]. A study by Longhurst et al. comparing elite endurance athletes and weightlifters reported that LVM increased in both groups compared with the control groups (amateur endurance athletes and weightlifters). However, he concluded that LVM increased more in elite endurance athletes than in elite weightlifters [11]. Therefore, research has shown that chronic exercise generally increases the aerobic ability of muscles and reduces CO2 at rest. These changes are associated with a slow heartbeat and increased myocardial contractility. The increased muscle oxygenation and hypertrophy with RE leads to higher oxygen perfusion [12]. Therefore, the increased amount of venous blood returning to the heart increases cardiac contractility via the Starling effect, which also increases the amount of blood leaving the heart. This resulting volume overload causes concentric cardiac remodeling in the heart muscle of resistance exercisers [13].

In the literature, cardiac morphology is affected by many exercise-related variables. Although there are many studies examining the effects of exercises such as RE on the heart [14–16], the number of studies examining the changes in LV diastolic and systolic parameters and left atrial (LA) mechanical function in individuals performing cardio exercises on different equipment in fitness centers is limited. Therefore, this study aimed to examine the effects of chronic RE and combined exercise (CE) on cardiac morphologies and to provide some suggestions for updating training content.

## Method

### Participants

The research sample was selected by simple random sampling method from the participants attending fitness centers [17]. The differences between the left ventricular end-diastolic diameter (LVEDV) values (experimental group: 65 ± 5 mL/m^2^, control group: 60 ± 5.2 mL/m^2^) in the results of the study conducted by Hassanpour Dehkordi and Khaledi Far were taken to determine the minimum sample size in the study [2]. In the power analysis performed according to the results of this study (α = 0.05 1-β(power) = 0.80, actual power = 81.9) and the effect size was taken as 1.05, it was determined that there should be at least 16 subjects for each group in our study. The study included volunteers who had been attending a fitness center regularly (at least three days per week) for at least 12 months. Participants with hypertension, tachycardia or bradycardia, thyroid disease, heart valve disease, previous surgery for a heart problem, and any comorbidities were excluded, as well as athletes taking caffeine, ephedrine, anabolic steroids, beta-androgenic agonists for performance enhancement. Participants who were found to have a sleep disorder during the study, who did not comply with the researcher’s instructions, or who were found to have health problems during testing were excluded from the study. Accordingly, 42 male amateur participants doing resistance exercises who met the inclusion and exclusion criteria were included in the study. Participants were divided into two groups according to the type of training: the RE group (*n* = 26), which performed only resistance exercises, and the CE group (*n* = 16), which combined cardio and resistance exercises. Participants’ time spent in sport (sport age), weekly exercise frequency, and exercise volume were recorded. RE group (sports age: 5.34 ± 7.31 years, weekly exercise frequency: 4.46 ± 1.36 days, daily training volume: 84.03 ± 17.88 s) and CE group (sports age: 7.15 ± 9.47 years, weekly exercise frequency: 4.06 ± 1.12 days, daily training volume: 84.31 ± 28.34 s) sports age, weekly training frequency and training volumes were similar (Table 1).

**Table 1:** Descriptive statistics of participants

Parameters	RE Group (*N* = 26)	CE Group (*N* = 16)	*p*
Age (Year)	27.03 ± 9.88	28.00 ± 11.52	0.775
Weight (kg)	81.53 ± 12.24	78.12 ± 12.53	0.977
Height (cm)	178.61 ± 4.85	178.56 ± 6.97	0.390
BMI (kg/m^2^)	25.50 ± 3.29	24.38 ± 2.57	0.253
BSA	2.00 ± 0.16	1.92 ± 0.28	0.245

BMI: Body Mass Index, BSA: Body Surface Area, RE: Resistance Exercise, CE: Combined Exercise

Institutional Review Board Statement: This study was conducted at Bandırma Onyedi Eylul University Health Sciences Institute Non-Interventional Ethics Committee, according to the principles outlined Declaration of Helsinki. Ethics committee approval was obtained from Bandırma Onyedi Eylul University Health Sciences Institute Non-Interventional Ethics Committee Ethics Committee (number: 2022 − 172). All experimental protocols were approved by the Institute’s Clinical Research Ethics Committee. Informed consent was obtained from all subjects and/or their legal guardian(s) agreed to participate in this study.

### Study design

In this study, the quantitative data collection technique known as the cross-sectional method was employed. This method involves measuring or observing specific behaviors of individuals from various age groups simultaneously, within the same time frame [18]. The RE group stated that they did not include cardio exercises (treadmill, elliptical trainer, vertical and horizontal cycling exercises, etc.) in their exercise program. The group CE stated that it includes at least 150 min of cardio exercises per week in addition to resistance exercises. They were asked to attend fully provided they slept at least 8 h before the test and ate at least 2 h before. Before the test, participants were informed that they should not engage in high-intensity sports and should not consume substances such as alcohol and caffeine. After the demographic information and training content of the participants were determined, they were taken to the cardiology department for analysis. After the necessary rest period was given, the DBP, SBP, and HR data of the participants were recorded. ECHO measurements were then performed by the cardiologist.

### Training characteristics of RE and CE groups

The RE group worked with free weights and machines as part of resistance exercises. All participants set their own 1 repetition maximum (1-RM). Each participant performed 6–12 repetitions*4 sets with 60–80% of 1-RM. The CE group included cardio exercises for 15–30 min in addition to RE exercises. Within the scope of these exercises, they stated that they worked between 60 and 70% of their HR on the treadmill, horizontal and vertical bicycle. They stated that they performed resistance exercises at the same standards as the RE group (60–80% 1-RM, 6–12 repetitions*4 sets). The exercise programs of the participants were prepared and followed by the expert trainer in the fitness center.

### Data Collection

#### Body surface area

After determining the demographic characteristics of the participants, such as age, height, and weight, body mass index (BMI) was calculated using the formula body weight (kg)/height squared (m²). Body surface area (BSA); Calculated according to the formula: Body weight (kg)^0.425^ ×Height (cm)^0.725^ × 0.007184 [19].

### Echocardiographic examinations

ECHO evaluations were made by a specialist cardiologist in the cardiology clinic. All tests were performed at the same time of day (morning). Participants were asked to take eight hours of sleep before the measurements. At least 3 h before the test, participants were warned not to use food, drink, performance-enhancing products, or products containing caffeine other than water. All ECHO examinations were performed with the Vivid T8 device and the 3ScRS transducer. All measurements were performed following the recommendations of the American Society of Echocardiography Guidelines [20]. Echocardiographic images and recordings were obtained in parasternal long axis, apical four-chamber, and apical two-chamber views in the left decubitus position and at rest [21]. The following 2-dimensional and M-mode echocardiographic parameters were measured: aortic diameters both in systole (ADs) and diastole (ADd), left ventricular end-diastolic diameter (LVDd, mm), left ventricular end-systolic diameter (LVDs), interventricular septal thickness (IVST), and posterior wall thickness (PWT).

Left ventricular end-diastolic volume (LVEDV), left ventricular end-systolic volume (LVESV), stroke volume (SV), and ejection fraction (EF) were measured in the apical four-chamber view by the modified Simpson method [22]. Pulsed wave (PW) and early diastolic flow velocity (E), late diastolic flow parameter (A), E/A ratio, ejection time (ET), isovolumic relaxation time (IVRT), isovolumic contraction time (IVCT), and transmissible flow parameters were measured. To determine the group with stage 2 diastolic dysfunction (pseudonormal), patients performed the Valsalva maneuver, and the measurement was repeated. Tissue Doppler imaging of annulus motion was measured from lateral mitral annulus and peak early systolic (Sm), peak early diastolic (Em), and peak late diastolic (Am) velocities.

### Tissue doppler imaging (TDI)

TDI was performed from the LV lateral wall in apical 4-chamber views from the mitral lateral annulus. A clear image signal was obtained by adjusting the TDI filter and Nyquist cutoff to a value of 16–20 cm/s. The left ventricular mass index (LVM-I) was calculated according to the Devereux formula [23]. Aortic systolic and diastolic diameters were obtained from the recording made after the insertion of the M-mode rod through the area of the ascending aorta 3 cm distal to the aortic valve. Systolic and diastolic diameters were measured in the area corresponding to the R peak of the ECG from the point of maximum forward motion in the aortic curve. The measurements were repeated in three heartbeats and the average value was determined [24]. Aortic strain (AS) and aortic distensibility (AD) were used as aortic elasticity parameters. The following formulas were used to calculate these parameters [25]:

$$\eqalign{{\rm{Aortic }}\,{\rm{Strain}}\,{\rm{ }}\left( {\rm{\% }} \right){\rm{ }}\,{\rm{ = }}\, & {\rm{ }}\left( {{\rm{systolic}}\,{\rm{ diameter}}\, - \,{\rm{diastolic }}\,{\rm{diameter}}} \right)\, \cr & \times \,{\rm{ 100 }}\,{\rm{/}}\,{\rm{diastolic}}\,{\rm{ diameter}} \cr}$$

$$\eqalign{{\rm{Distensibility}}\,{\rm{ }} & \cr & \left( {10\, - \,6.cm\, - \,2.dyn\, - \,1} \right){\rm{ }}\, \cr & = \,{\rm{ }}2\,{\rm{ }}\left( {{\rm{aortic}}\,{\rm{ strain }}} \right){\rm{ }}\, \cr & /\,{\rm{ }}\left( {{\rm{systolic }}\,{\rm{pressure}}\, - \,{\rm{diastolic}}\,{\rm{ pressure}}} \right) \cr}$$

Left atrial volumes were calculated from apical four-chamber and two-chamber views using the biplane field length method. Maximum left atrial volume (LAVmax) was measured at the time when the mitral valve was fully open, minimum left atrial volume (LAVmin) was measured at the time when the mitral valve was fully closed, and left atrial volume before systole was measured at the onset of the p wave (LAVp) on the electrocardiogram. All measurements were repeated during three consecutive heartbeats and averaged. The LAVmax index was determined by dividing the maximum volume of the left atrium (LAVmax) by the body surface area. All volumes were then corrected by division by the LAVmax index. Left atrial functions were determined according to the following formula [26].

$$\eqalign{{\rm{ LA}}\,{\rm{ passive}}\,{\rm{ emptying}}\,{\rm{ volume}}\,{\rm{ }}\left( {{\rm{LAPEV}}} \right){\rm{ }} & \, \cr & {\rm{ = }}\,{\rm{ LAV}}\,{\rm{ max}}\,{\rm{ -}}\,{\rm{ LAVp}} \cr}$$

$$\eqalign{{\rm{ LA }}\,{\rm{passive }}\,{\rm{emptying }}\,{\rm{fraction}}\,{\rm{ }}\left( {{\rm{LAPE}}F} \right){\rm{ }}\, & \cr & = \,{\rm{ LAPEV}}\,{\rm{ }}/\,{\rm{ LAVmax}} \cr}$$

$$\eqalign{{\rm{LA }}\,{\rm{active}}\,{\rm{ emptying}}\,{\rm{ volume }}\,\left( {{\rm{LAAEV}}} \right){\rm{ }}\, & \cr & {\rm{ = }}\,{\rm{ LAVp }}\,{\rm{-}}\,{\rm{ LAVmin}} \cr}$$

$$\eqalign{{\rm{LA}}\,{\rm{ active }}\,{\rm{emptying}}\,{\rm{ fraction}}\,{\rm{ }}\left( {{\rm{LAAEF}}} \right){\rm{ }} & \, \cr & {\rm{ = }}\,{\rm{ LAAEV }}\,{\rm{/}}\,{\rm{ LAVp}} \cr}$$

$$\eqalign{{\rm{LA}}\,{\rm{ total }}\,{\rm{emptying }}\,{\rm{volume}}\,{\rm{ }}\left( {{\rm{LATEV}}} \right) & \cr & {\rm{ }}\, = {\rm{ }}\,{\rm{LAVmax }}\, - \,{\rm{ LAVmin}} \cr}$$

$$\eqalign{{\rm{ LA }}\,{\rm{total}}\,{\rm{ emptying}}\,{\rm{ fraction}}\,{\rm{ }}\left( {{\rm{LATEF}}} \right){\rm{ }} & \cr & \,{\rm{ = }}\,{\rm{ LATEV }}\,{\rm{/}}\,{\rm{ LAVmax}} \cr}$$

$$\eqalign{{\rm{ Conduction volume}}\,{\rm{ }}\left( {{\rm{CV}}} \right){\rm{ }} & \, \cr & = \,{\rm{ Left}}\,{\rm{ ventricular}}\,{\rm{ stroke }}\,{\rm{volume }}\, \cr & - \,{\rm{ }}\left( {{\rm{LAVmax}}\, - \,{\rm{LAVmin}}} \right) \cr}$$

### Intraobserver and interobserver variability

The absolute mean difference ± SD between measurements within a single observer and between two observers for ADs, 2D STI, and LS were 1.1 ± 1.2% and 1.4 ± 1.4%, respectively. The intraobserver and interobserver Intraclass Correlation Coefficients (ICCs) were 0.877 and 0.865, respectively.

### Blood pressure

Participants’ blood pressure was measured by the cardiologist after 9 min of complete passive rest. Systolic and diastolic blood pressure were measured with a stethoscope and a sphygmomanometer (Erka Perfect Aneroid / Germany) [27].

### Statistical analysis

Data were reported as mean ± SD. Data analysis was carried out by the SPSS (version 25.0) program. The Shapiro-Wilk test stated the data were normally distributed. Also, according to Mauchly’s test, variances were determined to be homogeneous for all parameters (*p* > .05). Therefore, sphericity assumed values were taken into account. In the study, an Independent Sample T-test was used for paired group comparisons. In the research, graphical analyses were made using the GraphPad 9 program. The Cohen’s D effect size (ES) was performed to determine the effect magnitude through the difference of two means divided by the standard deviation from the data, and the following criteria were used: ES: 0.2 was accepted as small, 0.5 as medium, and 0.8 as large [28]. The significance level was interpreted according to *p* < .05.

The statistical analyses of this study were conducted using the Benjamini-Hochberg procedure to control the false discovery rate (FDR) that may result from the results of multiple comparisons. This procedure is a correction method developed to keep the probability of falsely rejecting multiple hypothesis tests low [29]. In this context, p-values were ranked at the end of the tests. Starting from the lowest p-value, the formula:

1 $$BHCi\, = \,(i\,/\,n)\, \times \,\alpha$$

was used for each p value. This procedure was used to limit possible false positives in the results and aimed to minimize the increased risk of type-1 error due to multiple comparisons.

## Results

Table 1 shows the descriptive information of the participants. Accordingly, there was no difference between RE’s age (27.03 ± 9.88 years), weight (81.53 ± 12.24 kg), height (178.61 ± 4.85 cm), BMI (25.50 ± 3.29 kg/m^2^), and BSA (2.00 ± 0.16) and CE’ age (28.00 ± 11.52 years), weight (78.12 ± 12.53 kg), height (178.56 ± 6.97 cm), BMI (24.38 ± 2.57 kg/m^2^), and BSA (1.92 ± 0.28) (*p* > .05).

Table 2 shows the statistical results of the participants’ SBP, DPB, HR, and routine ECHO parameters. Accordingly, LVEDD (*p* = .008, t = 2.805, ES: 0.84), SV-I (*p* = .048, t = 2.039, ES: 0.97), and LVEDV (*p* = .020, t = 2.415, ES: 0.75) in the RE group was significantly higher than the CE group. However, SBP, DBP, HR, ADs, ADd, LVESD, LVESV, IVST, and PWT values were similar between the two groups (*p* > .05).

**Table 2:** Examination of routine ECHO parameters on the cardio exercise status of the participants

Parameters	RE Group M ± S.D.	CE Group M ± S.D.	t	ES	*p*
SBP (mmHg)	124 ± 11	124 ± 14	0.063	0.01	0.950
DBP (mmHg)	73 ± 10	75 ± 12	-0.595	0.18	0.555
HR (beats)	80 ± 9	77 ± 12	1.043	0.31	0.303
ADs (mm)	27.46 ± 3.59	27.43 ± 4.36	0.597	0.07	0.985
ADd (mm)	22.42 ± 3.43	24.12 ± 5.73	0.149	0.36	0.235
LVEDD (mm)	49.80 ± 4.48	45.00 ± 6.64	2.805	0.84	0.008*
LVESD (mm)	32.69 ± 5.40	32.25 ± 7.62	0.086	0.06	0.827
LVEDV (mL/m^2^)	121.57 ± 23.02	102.68 ± 27.04	2.415	0.75	0.020*
LVESV (mL/m^2^)	43.00 ± 14.53	40.81 ± 14.33	0.476	0.15	0.637
SV-I (mL/m^2^)	77.61 ± 12.16	64.81 ± 13.96	2.039	0.97	0.048*
IVST (mm)	10.07 ± 1.49	10.56 ± 1.75	0.958	0.30	0.344
PWT (mm)	9.34 ± 1.91	9.43 ± 1.96	0.149	0.04	0.883

ADd: Aortic diameter diastole, ADs: Aortic diameter systole, DBP: Diastolic Blood Pressure, IVST: Interventricular septal thickness, LVEDD: Left ventricular end-diastolic diameter, LVESD: Left ventricular end-systolic diameter, LVDEV: Left ventricular end-diastolic volüme, LVESV: Left ventricular end-systolic volume, PWT: Posterior wall thickness, SBP: Systolic Blood Pressure, SV-I: Stroke Volume Index, RE: Resistance Exercise, CE: Combined Exercise

Table 3 shows the ECHO results of the systolic and diastolic functions of the participants. According to this, there was no difference between groups in terms of E, A, ET, IVCT, IVRT, Em, Am, Sm, LVEF, E/A ratio, and E/Em among participants (*p* > .05).

**Table 3:** Left ventricular systolic and diastolic functions of the participants

Parameters	RE Group M ± S.D.	CE Group M ± S.D.	t-value	ES	*p*
LVM-I (g/m^2^)	89.03 ± 18.00	83.93 ± 32.85	0.249	0.19	0.519
E (m/s)	1.35 ± 1.96	0.76 ± 0.14	1.540	0.42	0.136
A (m/s)	0.95 ± 1.35	0.52 ± 0.11	1.592	0.44	0.124
ET (ms)	287.00 ± 23.61	294.06 ± 30.96	0.835	0.25	0.409
IVCT (ms)	69.96 ± 13.48	75.62 ± 15.17	1.260	0.39	0.215
IVRT (ms)	66.65 ± 7.29	70.43 ± 13.07	-1.207	0.35	0.301
Em (cm/s)	14.11 ± 2.58	14.81 ± 2.71	0.834	0.26	0.409
Am (cm/s)	8.34 ± 2.05	8.81 ± 2.85	0.614	0.18	0.542
Sm (cm/s)	9.65 ± 1.89	9.31 ± 2.49	0.502	0.15	0.619
LVEF (%)	65.50 ± 7.10	64.00 ± 4.35	0.849	0.25	0.401
E/A Ratio	1.46 ± 0.36	1.51 ± 0.38	0.416	0.13	0.680
E/Em Ratio	0.10 ± 0.15	0.05 ± 0.00	1.622	0.13	0.117

A: Late diastolic flow parameter Am: Peak late diastolic, Em: Peak early diastolic, ET: Ejection time, E: Early diastolic flow velocity, IVCT: Isovolumic contraction time, IVRT: Isovolumic relaxation time, LVEF: Left ventricular ejection fraction, LVM-I: Left Ventricular Mass Index, Sm: Peak early systolic, RE: Resistance Exercise, CE: Combined Exercise

Table 4 shows the participants’ atrial functions. Accordingly, CV -I (*p* = .008, t = 2.797, ES:0.87) was higher in the RE group than in the CE group. However, LAAEV-I scores were higher in the CE group (*p* = .031, t=-2.230, ES: 0.67). There were no differences between groups in LAPEV-I, LAPEF-I, LAAEF-I, LATEV-I, LATEF-I, LAmin-I, LAP -I, LAmax-I, LVM-I, and AD scores (*p* > .05).

**Table 4:** ECHO results of left atrium functions and aortic stiffness parameters

Parameters	RE Group M ± S.D.	CE Group M ± S.D.	t-value	ES	*p*
LAPEV-I (mL/m^2^)	3.25 ± 0.98	3.11 ± 1.44	0.358	0.11	0.723
LAPEF-I (mL/m^2^)	0.20 ± 0.06	0.21 ± 0.08	− 0.304	0.14	0.763
LAAEV-I m(L/m^2^)	1.56 ± 0.53	1.99 ± 0.73	-2.230	0.67	0.031*
LAAEF-I (mL/m^2^)	0.20 ± 0.05	0.22 ± 0.04	-1.151	0.44	0.257
LATEV-I (mL/m^2^)	4.42 ± 1.08	5.51 ± 2.88	-1.743	0.50	0.166
LATEF-I(mL/m^2^)	0.32 ± 0.05	0.35 ± 0.36	-1.338	0.11	0.188
LAmin-I (mL/m^2^)	4.67 ± 1.30	5.06 ± 1.60	− 0.010	0.26	0.390
LAmax-I (mL/m^2^)	13.56 ± 2.45	15.05 ± 4.35	-1.258	0.42	0.222
LAP-I (mL/m^2^)	7.79 ± 1.58	8.87 ± 2.05	-1.912	0.59	0.063
CV-I (mL/m^2^)	34.36 ± 6.06	28.69 ± 6.87	2.797	0.87	0.008*

CV: Conduit Volume, LAAEF: Left Atrium Active Ejection Fraction Index, LAAEV: Left Atrium Active Ejection Volume Index, LAPEF: Left Atrium Passive Ejection Fraction Index, LAPEV-I: Left Atrium Passive Ejection Volume Index, LATEF-I: Left Atrium Total Ejection Fraction Index, LATEFSV-I: Left Atrium Total Ejection Fraction Index, RE: Resistance Exercise, CE: Combined Exercise

Table 5 shows the results of the aortic stiffness and flexibility of the participants. Accordingly, the results of AS (*p* = .032, t = 2.217, ES:0.75) were higher in the RE group than in the CE group. AD was similar between the two groups (*p* > .05).

**Table 5:** Comparison of participants’ aortic strain and distensibility

Parameters	RE Group M ± S.D.	CE Group M ± S.D.	t-value	ES	*p*
AS (%)	23.06 ± 8.11	17.38 ± 7.84	2.217	0.75	0.032*
AD (mmHg^− 1^.10^–3^)	91.11 ± 13.18	87.09 ± 19.65	0.795	0.28	0.431

AD: Aortic Distensibility, AS: Aortic Strain, RE: Resistance Exercise, CE: Combined Exercise

**Fig. 1:**
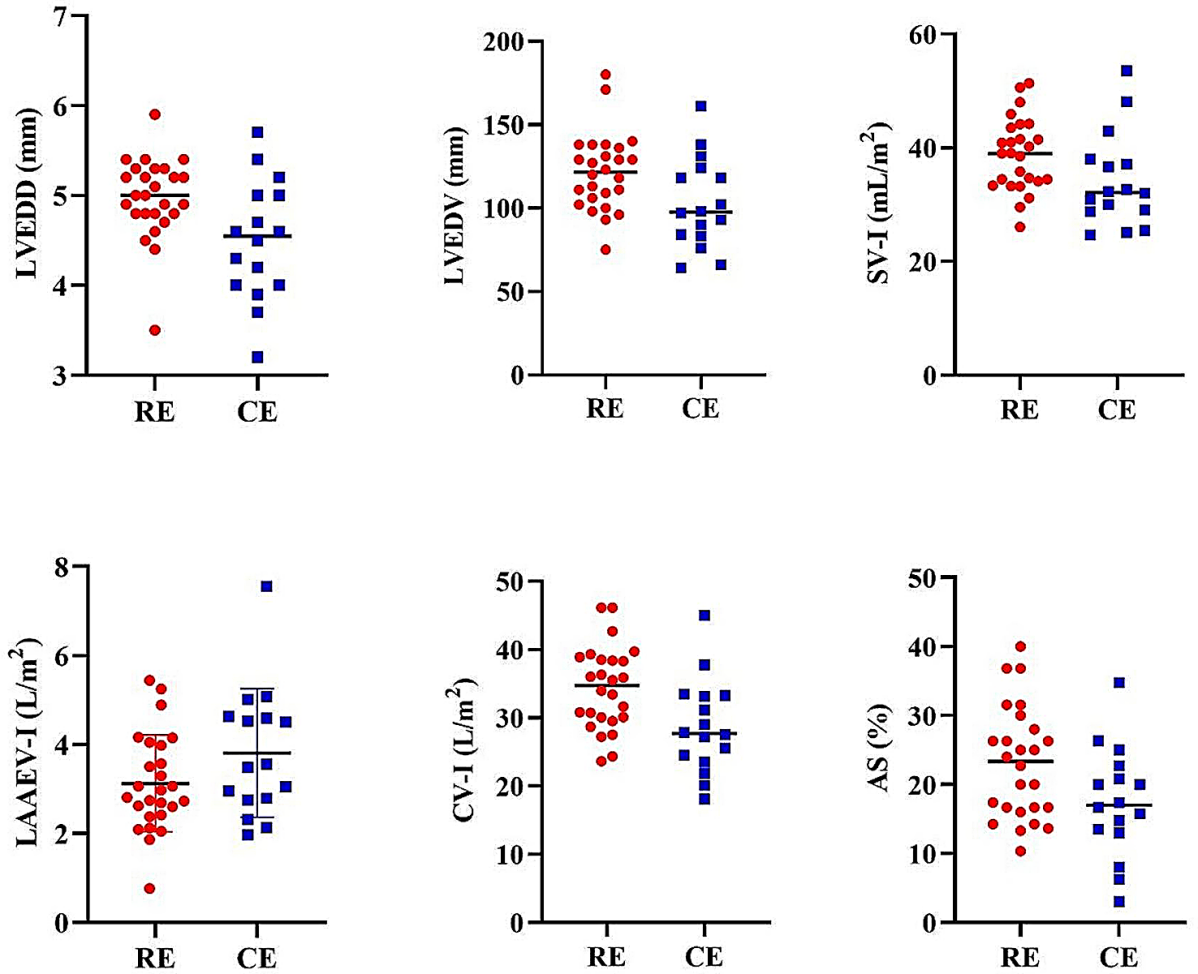
Differences between ECHO parameters of RE and CE groups

Figure 1 shows the parameters that differed in the ECHO results of the RE and CE groups. Accordingly, LVEDD (*p* = .008, t = 2.805, ES: 0.84), LVEDV (*p* = .020, t = 2.415, ES: 0.75), SV-I (*p* = .048, t = 2.039, ES: 0.97), CV -I (*p* = .008, t = 2.797, ES:0.87), and AS (*p* = .032, t = 2.217, ES:0.75) were significantly higher in the RE group than in the CE group. However, LAAEV-I scores were higher in the CE group (*p* = .031, t=-2.230, ES: 0.67).

## Discussion

In our study, the results for LVDd, LVEDV, SV -I, CV -I, and AS were higher in the RE than in CE. Also, LAAEV was higher in CE. From a physiological point of view, long-term hemodynamic changes during exercise induce both LV internal size (LV dilation) and LV hypertrophy to normalize LV wall tension. LV dilation and LV hypertrophy may be marked enough to mimic a pathological condition, but LV systolic and diastolic functions are normal or even above normal, which is a very natural feature of the athlete’s heart [30].

Chronic exercise is known to affect systolic [2] and diastolic [31] parameters. The changes in these parameters vary depending on the intensity, duration, and type of exercise [32]. During heavy resistance exercise, there is a significant increase in arterial blood pressure [33]. Cardiac output also increases. As a result, a slight increase in LVDd is observed [34]. It was found that systolic and diastolic parameters and cardiac output increased in individuals who combined exercises such as cycling, rowing, and cross-country skiing with resistance exercise. This excessive pressure exercise increases LVDd even more [35]. In a study by Keul et al., it was found that LVDd was higher in strength athletes than in long and middle-distance runners [36]. Although combined exercise should increase diastolic function more, it was found in our study that LVDd was higher in the RE group. In this case, it can be assumed that the intensity of cardio exercises performed in the CE group did not impose a burden on the heart. In a meta-analysis that examined the effects of aerobic, resistance, and combined exercise on cardiovascular factors, a systematic review study by Liang et al., the effects of aerobic, resistance, and combined exercises on cardiovascular risk factors were examined. According to the results of this study, it was reported that combined exercises were the most effective method in minimizing cardiac risk factors, while aerobic exercises had minimal effect. It was concluded that future studies need more study results for resistance exercises [37]. Evaluating the results of this study and the results of our study, we think that the structural changes expected as a result of endurance training in the heart did not occur, because the exercises performed by the group CE in addition to the RE exercises were of low intensity.

A relationship between the athlete’s heart and systolic and diastolic functions has been confirmed. The diastolic functions of the athlete’s heart are manifested by a low A wave, a high Em wave, and a lower E/Em ratio [38]. Elevated LV and confirmed SV -I for body surface area reflect systolic recovery [39]. A meta-analysis by Pluim et al. concluded that there was no significant difference in LVEF between endurance athletes and bodybuilders [40]. In our study, SV-I results were higher in the RE group than in the CE group. Although there was no difference between the groups in terms of LVEF, a positive correlation was found between LV and EF. In this case, it may show that the systolic parameters of the RE group are also positively affected. In a study by Seals et al., it was concluded that high-intensity exercise contributes to the development of LV in older elite athletes who have undergone endurance training [41].

In the study conducted by Andersen et al., systolic and diastolic indicators such as the E/Em ratio were analyzed as an estimate of Em, Sm, and left ventricular filling pressure [42], E increased significantly in both groups after sixteen weeks of football, and track and field training, noting that IVRT and Am scores decreased. However, it was observed that this change was greater in football players [43]. According to the results of our study, systolic and diastolic parameters (Em, Am, Sm, E/Em ratio) were similar to those reported in the literature and no significant difference was found between the two groups (RE, CE). Considering that resistance training increases aerobic capacity [1] and, accordingly, peak oxygen consumption and systolic parameters increase [44], the reason why the systolic and diastolic parameters of the RE group were better than those of the CE group is that the CE group could show that the groups training does not exert resistance on the heart.

With increasing age, significant changes occur in the cardiovascular system in apparently healthy people. There is thickening and hardening of the large arteries due to the loss of elastic fibers, collagen, and calcium deposits in the middle layer [45]. As a result of these arterial changes, SBP increases with age. Wall thickening occurs in the left ventricle due to cell hypertrophy [46]. LVD functions are preserved with age, the early diastolic filling rate decreases by 30–50% between the ages of 30–40 years [47]. These age-related cardiac changes can be ameliorated by physical activity. Acute and chronic exercise, on the other hand, elicit different responses in the cardiovascular system depending on age [48]. During acute exercise in healthy, sedentary individuals, SV increases by 20–30% [49]. As exercise intensity increases, LV develops in parallel [50]. In our study, it was found that SV -I values were significantly higher in the RE group. This indicates that in the RE group, due to muscle hypertrophy resulting from resistance exercise [51], the energy required by the muscles was provided by an increase in systolic parameters.

Aortic stiffness reflects the mechanical tension and elasticity of the aortic wall. Increased stiffness of the great arteries is an important determinant of adverse cardiovascular outcomes [25]. O’Rourke et al. reported that changes in vascular tone, smooth muscle cell hypertrophy and hyperplasia, and increased collagen synthesis lead to an increase in arterial stiffness [52]. In addition, in physically inactive individuals, large artery function in the cardiothoracic region may decrease with age [53]. However, aerobic exercise such as endurance sports is known to be an important method of preventing arterial stiffness [54]. Arterial stiffness did not change with less strenuous exercise [55]. The aorta is responsible for most of the total arterial stiffness [56]. Previous studies have cited AD and AS as the best predictors of subclinical arterial stiffness, and there is an inverse relationship between arterial stiffness and AS [57]. In our study, AS was significantly higher in the RE group. These results may resolve the uncertainties reported in previous studies [55] regarding arterial stiffness during resistance training.

In our study, there was no difference between the two groups in terms of left LVM and IVST. According to the Morganroth hypothesis, endurance training leads to eccentric LV hypertrophy (increase in LV cavity diameter at increased LVM) as a result of volume loading and increased diastolic wall stress [58]. Conversely, resistance training results in concentric LV hypertrophy (increased wall thickness with no change in cavity size) as a result of pressure loading and increased systolic wall stress [59]. Data from Italian screening programs reported LV dilatation and LV hypertrophy in a large cohort of young athletes studied by ECHO. Although LVDd varied widely, most athletes were above normal, whereas only a small percentage had an IVST > 12 mm [60]. Standard ECHO plays an important role in distinguishing physiological from pathological LV hypertrophy. In the 1309 athlete series in different disciplines, 55% of endurance athletes have increased LVDd and only 15% have a value > 60 mm, almost always with normal EF. Most of the 947 top athletes had an IVST ≤ 12 mm. Only 1.7% had an IVST > 13 mm (range = 13–16 mm). While an increase in LVM in athletes is usually associated with a normal EF at rest, HR may be normal or increased as a result of increased preload (LVDd) [61].

Previous studies examined left atrial (LA) size and athletic activity in athletes and found that left atrial enlargement depended on the type of exercise (the greatest change occurred with combined exercise: Endurance and strength exercises) [62]. Although the LA is known as a transport space that carries blood from the pulmonary veins to the LV during active and passive diastolic filling, it also has many physiological functions and acts as a volume sensor. It is well known that LA size is an important indicator of mortality and morbidity in cardiomyopathy, LV dysfunction, aortic stenosis, mitral regurgitation, and arrhythmias [63]. Like the LV in LA also remodels after endurance exercise [64]. However, an important aspect is that LA reserve helps modulate LV filling pressure both at rest and during exercise [65]. Although the mechanisms of atrial development are not fully known [66], some sources indicate that the LA develops anterior to the LV in response to exercise [67]. Pellicia et al. found a prevalence of LA enlargement in athletes, a slight increase in LA anteroposterior diameter in 18% of athletes, significant dilation in 2%, and a close relationship between LA diameter and LA cavity [68]. In a study by Lakatos et al. comparing left atrial morphology in elite athletes with sedentary subjects, it was found that LATEF and LAAEF were lower in athletes [69]. A recent meta-analysis also found that both LA reservoir and contractile functions were lower in athletes [70]. In our study, LAAEF was significantly higher in the CE group than in the RE group. When participants’ left atrial functions are assessed according to the results of the study by Lakatos et al’, the LAAEV results of the CE group show that they do not have the characteristics of an athlete’s heart compared with the literature. Although no significant difference was found, the LAP -I and LATEV-I results had a moderate effect size and were higher in the CE group.

Our research is a study in which training loads and exercise intensities were analyzed according to the participants’ statements. This is one of the important limitations of our study. Therefore, research can be conducted on how the cardiac remodeling of the participants is shaped after chronic exercise. Another important limitation of this study is that it is cross-sectional in nature using a single time point. Therefore, in future randomised controlled trials, the changes in resistance exercises and aerobic exercise on cardiac morphology can be analysed in detail.

## Conclusion

In conclusion, in our study, it was found that systolic and diastolic parameters were higher in the RE group than in the CE group. In addition, a significant increase in LAAEF in the CE group is indicative of an increase in active LA functions. Other findings on LA functions supported the increase in LAAEF in the CE group, although there was no significant difference. In addition, the higher systolic and diastolic parameters such as LVDd, LVEDV, SV -I, and CV -I in the RE group confirmed that the RE group had shown more athletes’ heart [71]. In the literature, combined exercise with resistance exercise is more effective on cardiac functions [33, 37, 72], but in our study, indices of cardiac functions were lower in the CE group than in the RE group. It is believed that this is because the CE group kept the exercise intensity of cardio training equipment such as treadmills, elliptical bikes, and vertical and horizontal bikes lower in the exercises they performed. For this reason, athletes who visit a fitness center can update their programs in the presence of a sports professional. In addition, the level of physical fitness of these people can be monitored at regular intervals and their development can be analyzed. Individuals who attend the fitness centre for different purposes can update their training programs and intensities according to the results of our research.

### Electronic supplementary material

Below is the link to the electronic supplementary material.

Supplementary Material 1

## Data Availability

Data are available for research purposes upon reasonable request to the corresponding author.
